# Supplementary material for: The impact of species-wide gene expression variation on Caenorhabditis elegans complex traits
Source: Nat Commun. 2022 Jun 16;13:3462. doi: 10.1038/s41467-022-31208-4 (PMC9203580; doi:10.1038/s41467-022-31208-4)
Supplement: Supplementary file 1 — Supplementary Information [file 41467_2022_31208_MOESM1_ESM.pdf]

Supplementary Information

The impact of species-wide gene expression variation on *Caenorhabditis elegans* complex traits

Gaotian Zhang[1], Nicole M. Roberto[1], Daehan Lee[1], Steffen R. Hahnel[1], and Erik C. Andersen[1,*]

1. Department of Molecular Biosciences, Northwestern University, Evanston, IL 60208, USA

Supplementary Figures

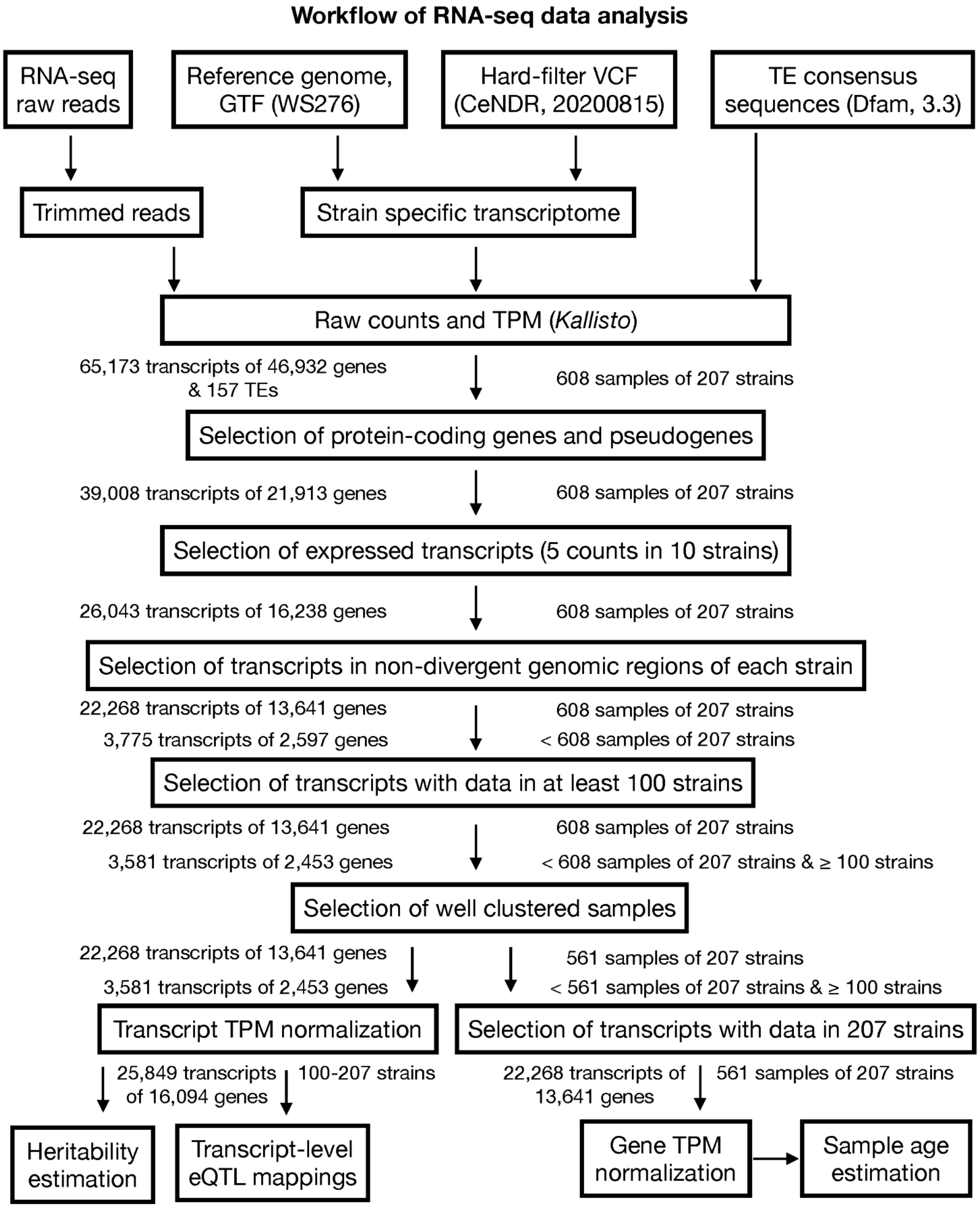

Supplementary Fig. 1

Workflow of RNA-seq data processing. The numbers of transcripts, genes, samples, and strains from each step to the next step are indicated on either side of the vertical arrows.

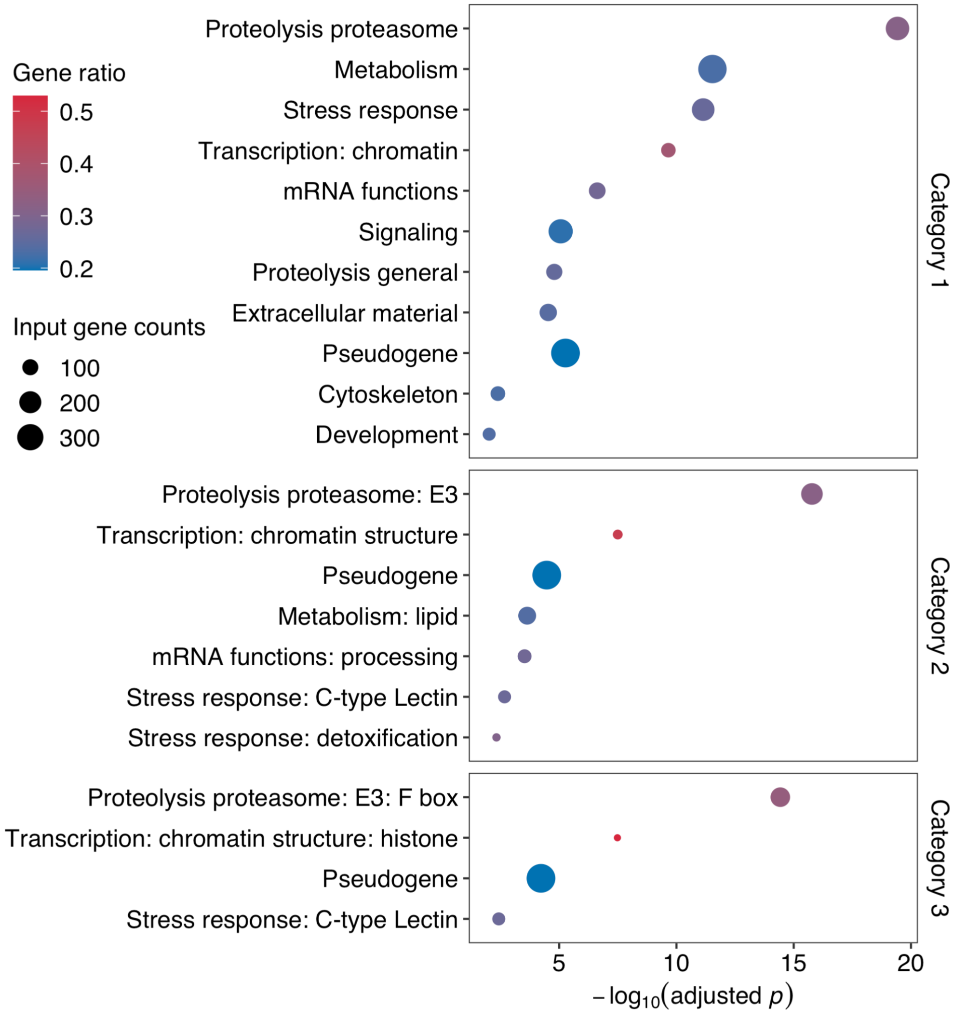

Supplementary Fig. 2

Gene set enrichment analysis for genes with transcript level eQTL. Enriched gene classes of broad and specific categories (Category 1 to 3)[1] are shown on the y axis. Bonferroni FDR corrected significance values using one-sided Fisher's Exact Test for gene set enrichment analysis are shown on the x axis. The sizes of the circles correspond to the input gene counts of the annotation and the colors of the circles correspond to the gene ratio of input gene counts to total gene counts of the annotation. Wormcat[1] provides enrichment results in broad categories (those in Category 1) and more specific categories (in Category 2 and 3). For example, the top enrichment in Category 1 is the proteolysis proteasome, more specifically, the ubiquitin ligases E3 of proteolysis proteasome in Category 2 and the F-box protein of ubiquitin ligases E3 of proteolysis proteasome in Category 3.

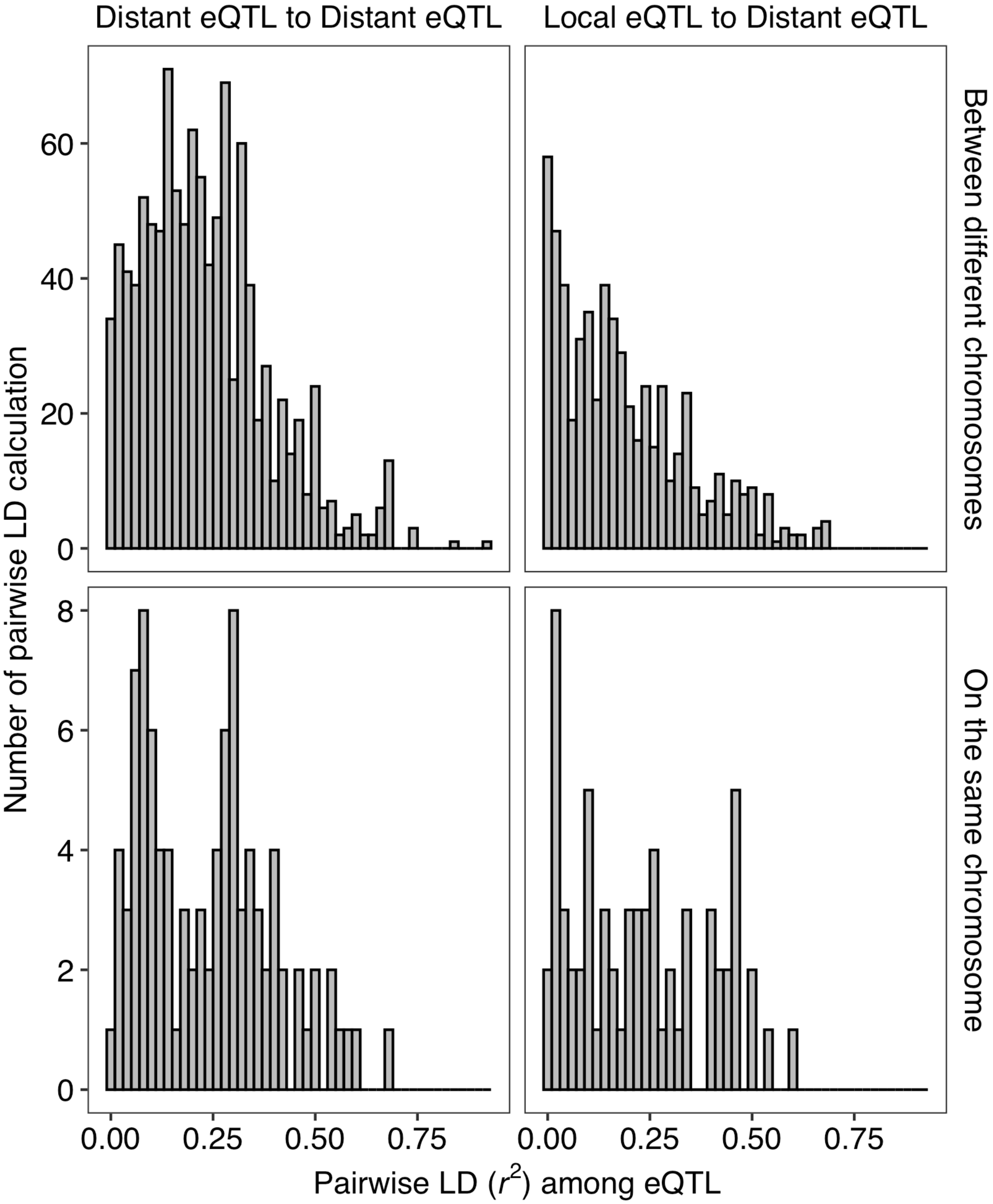

Supplementary Fig. 3

A histogram showing the distribution of linkage disequilibrium (LD) (r^2) (x-axis) among QTL of multiple eQTL of transcript expression traits. A total of 861 traits were found with multiple eQTL.

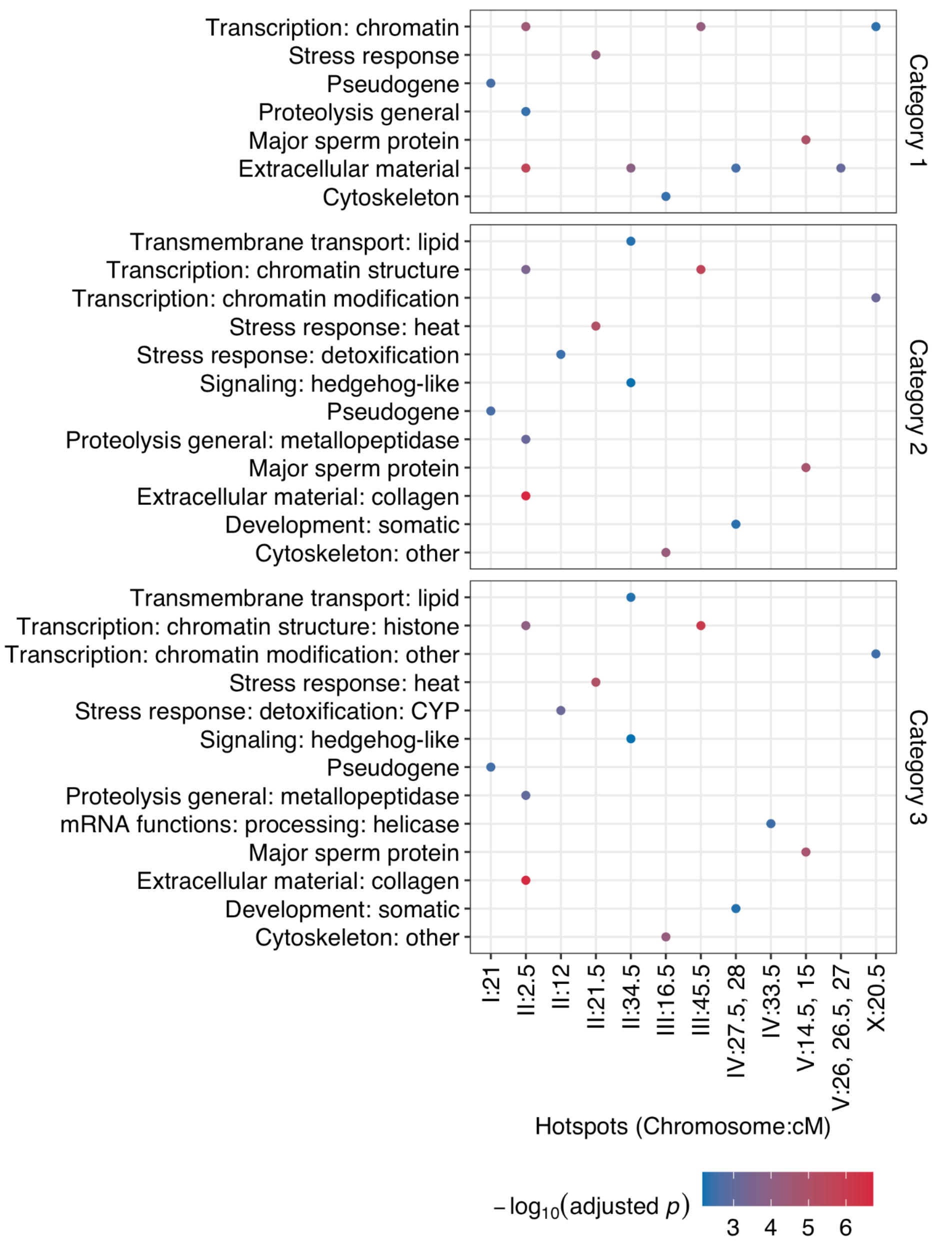

Supplementary Fig. 4

Gene set enrichment analysis for genes with transcript level distant eQTL in each hotspot. Broad and specific categories of enriched gene (Category 1 to 3)[1] are shown on the y axis. Distant eQTL hotspots with significant gene set enrichment are shown on the x axis. The colors of the circles correspond to Bonferroni FDR corrected significance values using one-sided Fisher's Exact Test. Only hotspots with significant enrichments were shown.

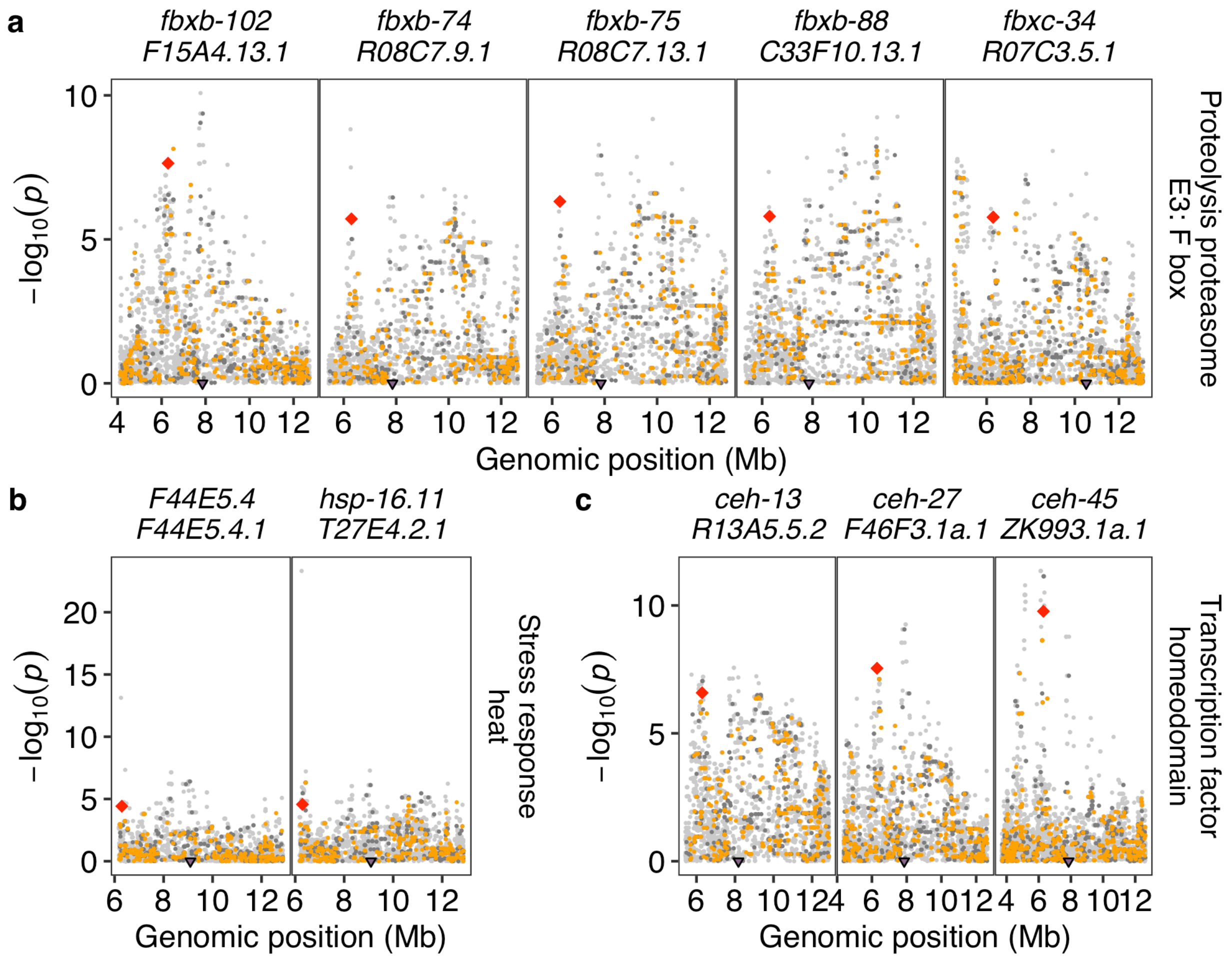

Supplementary Fig. 5

Fine mapping of 10 transcript expression traits with *hil-2* as a candidate gene in distant eQTL in the hotspot at 30.5-33 cM on chromosome IV is shown. The enrichment category for each panel was indicated on the right. F-box proteins (E3 ligase complex components), heat shock response, and transcription factors with homeodomains were the three enriched gene classes for genes in **a**, **b**, and **c**, respectively. Each dot represents a variant that is plotted with its genomic position (x-axis) against the -log10(*p*) values (y-axis). Purple triangles on the x-axis represent eQTL positions. Candidate variants in *hil-2* with negative BLOSUM scores are indicated as red diamonds. Other variants that are with negative BLOSUM scores, with non-negative BLOSUM scores or intergenic are colored orange, dark gray, and light gray, respectively. Gene names and transcript names of each trait are indicated above each panel. We used the *GWAS()* function in the R package *rrBLUP*[2] to perform the genome-wide mapping with the EMMA algorithm[3] (See Methods).

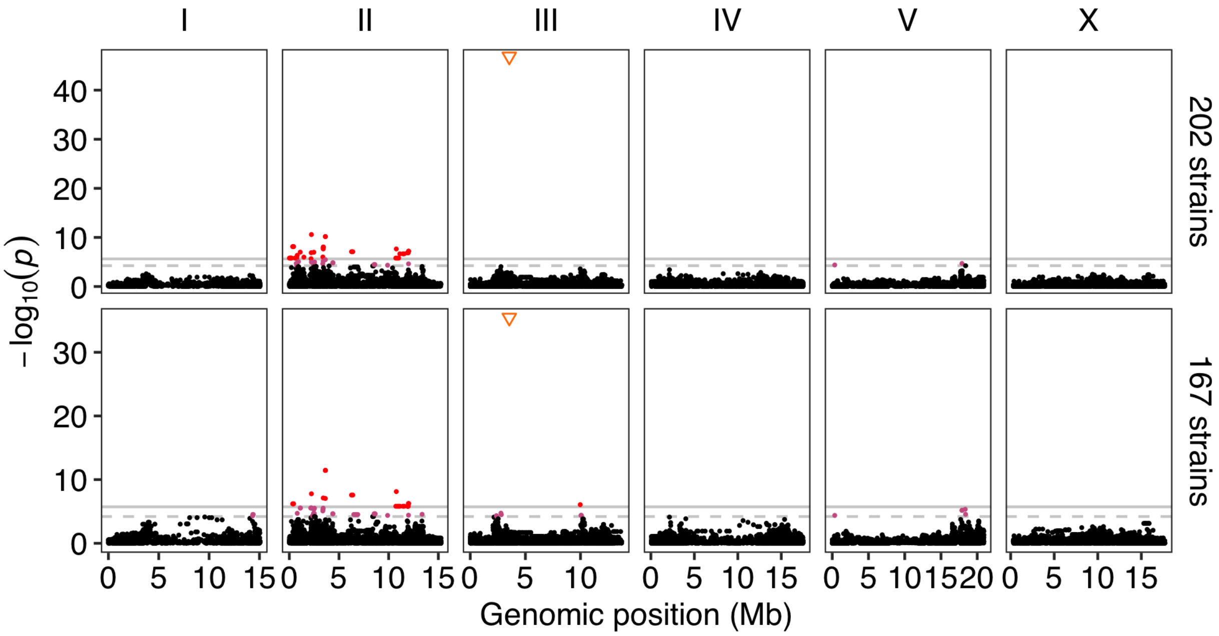

Supplementary Fig. 6

Manhattan plots indicating the GWA mapping result for animal length (q90.TOF) of 202 (top panel) and 167 (bottom panel) *C. elegans* wild strains in response to ABZ[4] are shown. Each point represents an SNV that is plotted with its genomic position (x-axis) against its -$\log_{10}(p)$ value (y-axis) from the GWA mapping. Real SNVs that pass the genome-wide EIGEN threshold (the dotted gray horizontal line) and the genome-wide Bonferroni threshold (the solid gray horizontal line) are colored pink and red, respectively. The pseudo SNV marker representing high allelic heterogeneity in the gene *ben-1* at position 3,539,640 on chromosome III is indicated as an orange inverted triangle. We used the *GWAS()* function in the R package *rrBLUP*[2] to perform the genome-wide mapping with the EMMA algorithm[3] (See Methods).

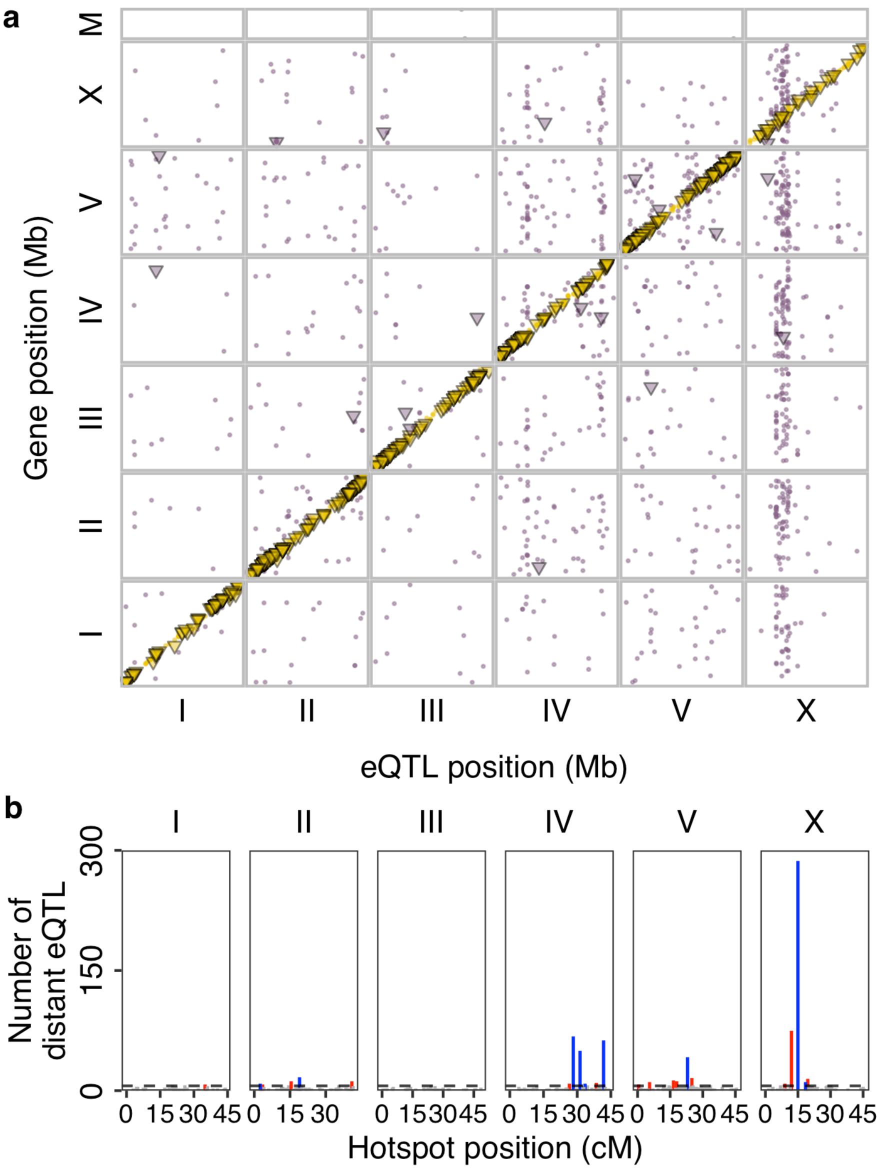

Supplementary Fig. 7

RIAILs eQTL. **a** The genomic locations of 2,387 eQTL peaks (x-axis) in the RIAILs eQTL studies[5,6] are plotted against the genomic locations of the 2,003 genes with expression differences (y-axis). Golden points or triangles on the diagonal of the map represent local eQTL. Purple points or triangles correspond to distant eQTL. Triangles represent eQTL that were also found in our study. **b** The number of distant eQTL (y-axis) in each 0.5 cM bin across the genome (x-axis) is shown. The horizontal dashed line indicates the threshold of six eQTL. Bins with six or more eQTL were identified as hotspots and are colored red or blue. Bins with fewer than six eQTL are colored gray. Blue bins represent hotspots that were also found in our study.

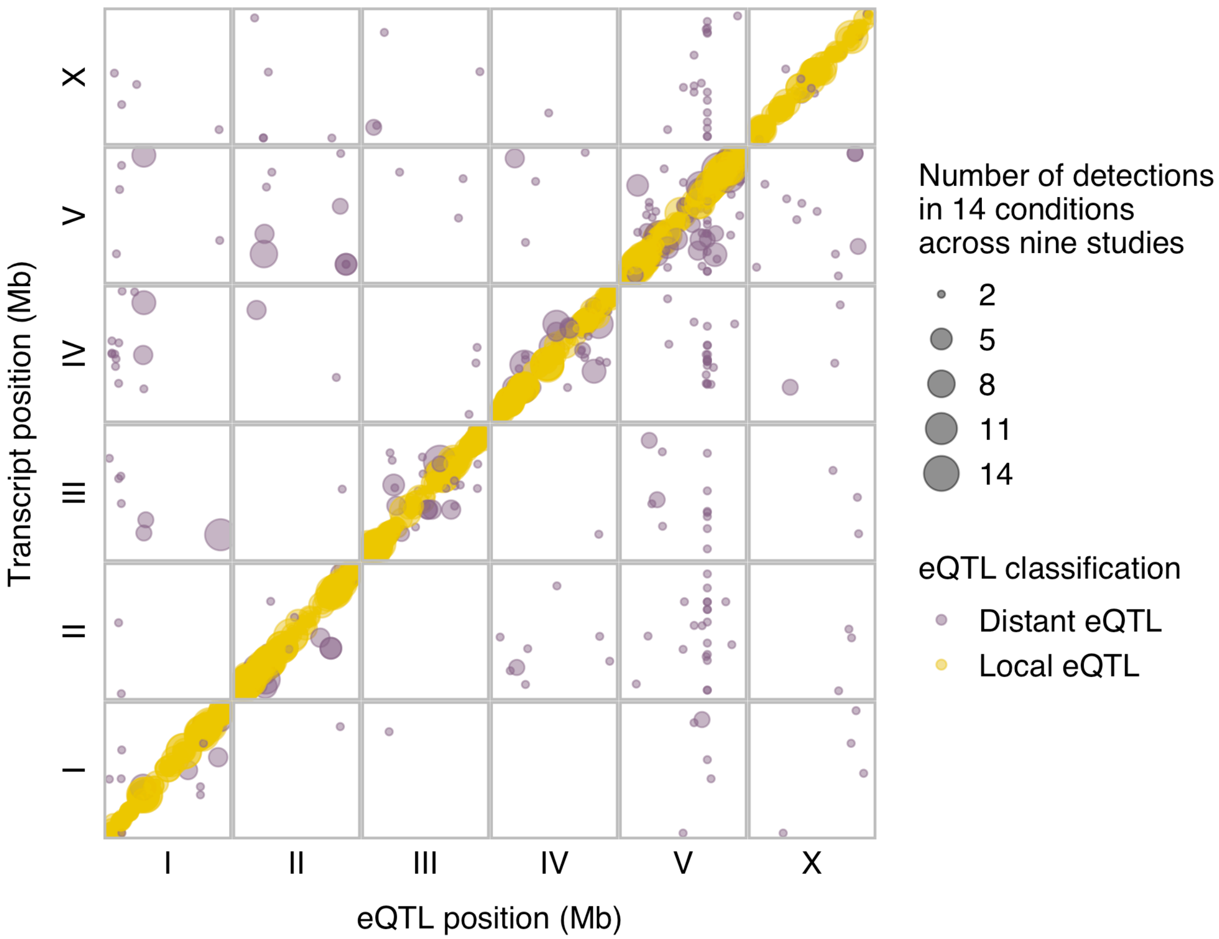

Supplementary Fig. 8

The genomic locations of 2,029 eQTL peaks (x-axis) that overlapped with eQTL detected in eight previous studies[5–13] are plotted against the genomic locations of the 1,993 transcripts of 1,625 genes with expression differences (y-axis). Golden points on the diagonal of the map represent local eQTL that colocalize with the transcripts that they influence. Purple points correspond to distant eQTL that are located further away from the transcripts that they influence. The size of each point represents the total number of detections for each eQTL in the 14 conditions of the nine studies, including this study.

References

1. Holdorf, A. D. *et al.* WormCat: An Online Tool for Annotation and Visualization of Caenorhabditis elegans Genome-Scale Data. *Genetics* **214**, 279–294 (2020).
2. Endelman, J. B. Ridge regression and other kernels for genomic selection with R package rrBLUP. *Plant Genome* **4**, 250–255 (2011).
3. Kang, H. M. *et al.* Efficient control of population structure in model organism association mapping. *Genetics* **178**, 1709–1723 (2008).
4. Hahnel, S. R. *et al.* Extreme allelic heterogeneity at a Caenorhabditis elegans beta-tubulin locus explains natural resistance to benzimidazoles. *PLoS Pathog.* **14**, e1007226 (2018).
5. Rockman, M. V., Skrovanek, S. S. & Kruglyak, L. Selection at linked sites shapes heritable phenotypic variation in C. elegans. *Science* **330**, 372–376 (2010).
6. Evans, K. S. & Andersen, E. C. The Gene scb-1 Underlies Variation in Caenorhabditis elegans Chemotherapeutic Responses. *G3* **10**, 2353–2364 (2020).
7. Li, Y. *et al.* Mapping determinants of gene expression plasticity by genetical genomics in C. elegans. *PLoS Genet.* **2**, e222 (2006).
8. Viñuela, A., Snoek, L. B., Riksen, J. A. G. & Kammenga, J. E. Genome-wide gene expression regulation as a function of genotype and age in C. elegans. *Genome Res.* **20**, 929–937 (2010).
9. Li, Y. *et al.* Global genetic robustness of the alternative splicing machinery in Caenorhabditis elegans. *Genetics* **186**, 405–410 (2010).
10. Sterken, M. G. *et al.* Ras/MAPK Modifier Loci Revealed by eQTL in Caenorhabditis elegans. *G3* **7**, 3185–3193 (2017).
11. Snoek, B. L. *et al.* Contribution of trans regulatory eQTL to cryptic genetic variation in C. elegans. *BMC Genomics* **18**, 500 (2017).
12. Snoek, B. L. *et al.* The genetics of gene expression in a Caenorhabditis elegans multiparental recombinant inbred line population. *G3* **11**, (2021).
13. Ben-David, E. *et al.* Whole-organism eQTL mapping at cellular resolution with single-cell sequencing. *Elife* **10**, (2021).
