# Supplementary material for: The impact of species-wide gene expression variation on Caenorhabditis elegans complex traits
Source: Nat Commun. 2022 Jun 16;13:3462. doi: 10.1038/s41467-022-31208-4 (PMC9203580; doi:10.1038/s41467-022-31208-4)
Supplement: Supplementary file 3 — Description of Additional Supplementary Files [file 41467_2022_31208_MOESM3_ESM.pdf]

Description of Additional Supplementary Files

File Name: Supplementary Data 1
Description: Expression abundances and heritabilities of 25,849 transcripts in 207 wild C. elegans strains

File Name: Supplementary Data 2
Description: GWA mapping results of 5,291 transcript expression traits, eQTL classification, and distant eQTL hotspots

File Name: Supplementary Data 3
Description: : Enrichment of genes with eQTL, distant eQTL in hotspots

File Name: Supplementary Data 4
Description: Fine mappings for distant eQTL in hotspots
