# Supplementary material for: The impact of species-wide gene expression variation on Caenorhabditis elegans complex traits
Source: Nat Commun. 2022 Jun 16;13:3462. doi: 10.1038/s41467-022-31208-4 (PMC9203580; doi:10.1038/s41467-022-31208-4)
Supplement: Supplementary file 5 — Reporting Summary [file 41467_2022_31208_MOESM5_ESM.pdf]

nature portfolio

Corresponding author(s): Erik C. Andersen

Last updated by author(s): May 29, 2022

Reporting Summary

Nature Portfolio wishes to improve the reproducibility of the work that we publish. This form provides structure for consistency and transparency in reporting. For further information on Nature Portfolio policies, see our Editorial Policies and the Editorial Policy Checklist.

Statistics

For all statistical analyses, confirm that the following items are present in the figure legend, table legend, main text, or Methods section.

n/a	Confirmed	
☐	☒	The exact sample size (*n*) for each experimental group/condition, given as a discrete number and unit of measurement
☐	☒	A statement on whether measurements were taken from distinct samples or whether the same sample was measured repeatedly
☐	☒	The statistical test(s) used AND whether they are one- or two-sided<br>*Only common tests should be described solely by name; describe more complex techniques in the Methods section.*
☐	☒	A description of all covariates tested
☐	☒	A description of any assumptions or corrections, such as tests of normality and adjustment for multiple comparisons
☐	☒	A full description of the statistical parameters including central tendency (e.g. means) or other basic estimates (e.g. regression coefficient) AND variation (e.g. standard deviation) or associated estimates of uncertainty (e.g. confidence intervals)
☐	☒	For null hypothesis testing, the test statistic (e.g. *F*, *t*, *r*) with confidence intervals, effect sizes, degrees of freedom and *P* value noted<br>*Give P values as exact values whenever suitable.*
☒	☐	For Bayesian analysis, information on the choice of priors and Markov chain Monte Carlo settings
☒	☐	For hierarchical and complex designs, identification of the appropriate level for tests and full reporting of outcomes
☐	☒	Estimates of effect sizes (e.g. Cohen's *d*, Pearson's *r*), indicating how they were calculated

Our web collection on statistics for biologists contains articles on many of the points above.

Software and code

Policy information about availability of computer code

Data collection	No software was used for data collection.
Data analysis	The RNA-seq mapping pipeline can be found at https://github.com/AndersenLab/PEmRNA-seq-nf. The mediation analysis pipeline can be found at https://github.com/AndersenLab/mediation_GWAeQTL. The code for generating all figures can be found at https://github.com/AndersenLab/WI-Ce-eQTL.<br><br>The pipeline cegwas2-nf (https://github.com/AndersenLab/cegwas2-nf)<br>The web-based tool WormCat (http://wormcat.com/)<br>The script TEconsensus (https://github.com/fansalon/TEconsensus)<br>fastp (v0.20.0)<br>FastQC (v0.11.8) analysis (http://www.bioinformatics.babraham.ac.uk/projects/fastqc)<br>BCFtools (v.1.9)<br>gffread (v0.11.6)<br>Kallisto (v0.44.0)<br>PLINK (v1.96)<br>R (v3.6.0)<br>R package sleuth (v0.30.0)<br>R package tximport (v1.10.1)<br>R package DESeq2 (v1.26.0)<br>R package RAPToR (v1.1.3)<br>R package phangorn (v2.5.5)

R package ape (v5.6)
R package ggtree (v1.14.6)
R package rrBLUP (v4.6.1)
R package RSpectra (v0.16.0) (https://github.com/yixuan/RSpectra)
R package correlateR (0.1) (https://github.com/AEBilgrau/correlateR)
R package genetics (v1.3.8.1.2) (https://cran.r-project.org/package=genetics)
R package sommer (v4.1.2)
R package lme4 (v1.1.21)
R package EnvStats (v2.3.1)
R package linkagemapping (v1.3) (https://github.com/AndersenLab/linkagemapping)
R package MultiMed (v2.6.0) (https://bioconductor.org/packages/release/bioc/html/MultiMed.html)
R package mediation (version 4.5.0)

1. Holdorf, A. D. et al. WormCat: An Online Tool for Annotation and Visualization of Caenorhabditis elegans Genome-Scale Data. Genetics 214, 279–294 (2020).
2. Chen, S., Zhou, Y., Chen, Y. & Gu, J. fastp: an ultra-fast all-in-one FASTQ preprocessor. Bioinformatics 34, i884–i890 (2018).
3. Li, H. A statistical framework for SNP calling, mutation discovery, association mapping and population genetical parameter estimation from sequencing data. Bioinformatics 27, 2987–2993 (2011).
4. Pertea, G. & Pertea, M. GFF Utilities: GffRead and GffCompare. F1000Res. 9, 304 (2020).
5. Bray, N. L., Pimentel, H., Melsted, P. & Pachter, L. Near-optimal probabilistic RNA-seq quantification. Nat. Biotechnol. 34, 525–527 (2016).
6. Purcell, S. et al. PLINK: a tool set for whole-genome association and population-based linkage analyses. Am. J. Hum. Genet. 81, 559–575 (2007).
7. Chang, C. C. et al. Second-generation PLINK: rising to the challenge of larger and richer datasets. Gigascience 4, 7 (2015).
8. Core Team, R. & Others. R: A language and environment for statistical computing. Vienna, Austria: R Foundation for Statistical Computing. Available (2013).
9. Pimentel, H., Bray, N. L., Puente, S., Melsted, P. & Pachter, L. Differential analysis of RNA-seq incorporating quantification uncertainty. Nat. Methods 14, 687–690 (2017).
10. Soneson, C., Love, M. I. & Robinson, M. D. Differential analyses for RNA-seq: transcript-level estimates improve gene-level inferences. F1000Res. 4, 1521 (2015).
11. Love, M. I., Huber, W. & Anders, S. Moderated estimation of fold change and dispersion for RNA-seq data with DESeq2. Genome Biol. 15, 550 (2014).
12. Bulteau, R. & Francesconi, M. Real age prediction from the transcriptome with RAPToR. bioRxiv 2021.09.07.459270 (2021) doi:10.1101/2021.09.07.459270.
13. Schliep, K. P. phangorn: phylogenetic analysis in R. Bioinformatics 27, 592–593 (2011).
14. Paradis, E. & Schliep, K. ape 5.0: an environment for modern phylogenetics and evolutionary analyses in R. Bioinformatics 35, 526–528 (2019).
15. Yu, G., Smith, D. K., Zhu, H., Guan, Y. & Lam, T. T. Ggtree : An r package for visualization and annotation of phylogenetic trees with their covariates and other associated data. Methods Ecol. Evol. 8, 28–36 (2017).
16. Endelman, J. B. Ridge regression and other kernels for genomic selection with R package rrBLUP. Plant Genome 4, 250–255 (2011).
17. Covarrubias-Pazaran, G. Genome-Assisted Prediction of Quantitative Traits Using the R Package sommer. PLoS One 11, e0156744 (2016).
18. Bates, D., Mächler, M., Bolker, B. & Walker, S. Fitting Linear Mixed-Effects Models Using lme4. Journal of Statistical Software, Articles 67, 1–48 (2015).
19. Millard, S. P. EnvStats: An R Package for Environmental Statistics. (Springer-Verlag New York, 2013).
20. Tingley, D., Yamamoto, T., Hirose, K., Keele, L. & Imai, K. mediation: R Package for Causal Mediation Analysis. Journal of Statistical Software, Articles 59, 1–38 (2014).

For manuscripts utilizing custom algorithms or software that are central to the research but not yet described in published literature, software must be made available to editors and reviewers. We strongly encourage code deposition in a community repository (e.g. GitHub). See the Nature Portfolio guidelines for submitting code & software for further information.

Data

Policy information about availability of data

All manuscripts must include a data availability statement. This statement should provide the following information, where applicable:

- Accession codes, unique identifiers, or web links for publicly available datasets
- A description of any restrictions on data availability
- For clinical datasets or third party data, please ensure that the statement adheres to our policy

The raw RNA-seq data generated in this study are available at the NCBI Sequence Read Archive (Project PRJNA669810). The raw expression counts and TPM quantified in this study are available at NCBI's Gene Expression Omnibus (Series GSE186719). The datasets for generating all figures can be found at https://github.com/AndersenLab/WI-Ce-eQTL.

The C. elegans reference genome (WS276) and the GTF file (WS276) were obtained from WormBase (ftp://ftp.wormbase.org//pub/wormbase/releases/WS276/species/c_elegans/PRJNA13758).
The hard-filtered isotype VCF (20200815 release) was obtained from CeNDR (https://www.elegansvariation.org/data/release/20200815).

The transposable element (TE) sequences of C. elegans were obtained from Dfam (release 3.3) (https://www.dfam.org/releases/Dfam_3.3/).

Previous C. elegans eQTL data were obtained from WormQTL2 (https://www.bioinformatics.nl/EleQTL/?mode=download) and the original papers:

1. Snoek, B. L. et al. WormQTL2: an interactive platform for systems genetics in Caenorhabditis elegans. Database 2020, (2020).
2. Rockman, M. V., Skrovanek, S. S. & Kruglyak, L. Selection at linked sites shapes heritable phenotypic variation in C. elegans. Science 330, 372–376 (2010).
3. Evans, K. S. & Andersen, E. C. The Gene scb-1 Underlies Variation in Caenorhabditis elegans Chemotherapeutic Responses. G3 10, 2353–2364 (2020).
4. Li, Y. et al. Mapping determinants of gene expression plasticity by genetical genomics in C. elegans. PLoS Genet. 2, e222 (2006).
5. Viñuela, A., Snoek, L. B., Riksen, J. A. G. & Kammenga, J. E. Genome-wide gene expression regulation as a function of genotype and age in C. elegans. Genome Res. 20, 929–937 (2010).
6. Li, Y. et al. Global genetic robustness of the alternative splicing machinery in Caenorhabditis elegans. Genetics 186, 405–410 (2010).
7. Sterken, M. G. et al. Ras/MAPK Modifier Loci Revealed by eQTL in Caenorhabditis elegans. G3 7, 3185–3193 (2017).
8. Snoek, B. L. et al. Contribution of trans regulatory eQTL to cryptic genetic variation in C. elegans. BMC Genomics 18, 500 (2017).
9. Snoek, B. L. et al. The genetics of gene expression in a Caenorhabditis elegans multiparental recombinant inbred line population. G3 11, (2021).
10. Ben-David, E. et al. Whole-organism eQTL mapping at cellular resolution with single-cell sequencing. Elife 10, (2021).

Field-specific reporting

Please select the one below that is the best fit for your research. If you are not sure, read the appropriate sections before making your selection.

☒ Life sciences ☐ Behavioural & social sciences ☐ Ecological, evolutionary & environmental sciences

For a reference copy of the document with all sections, see nature.com/documents/nr-reporting-summary-flat.pdf

Life sciences study design

All studies must disclose on these points even when the disclosure is negative.

Sample size	The processes of animal growth and harvesting were done during a large-scale GWA project on C. elegans variation to drugs in 2017. At that time, we had 249 isotype strains in the collection (CeNDR version 20170531). We aimed to test every isotype strain for drug response with at least three biological replicates and harvested synchronized worms in the control condition for RNA-seq. To prepare animals for RNA extraction, we grew approximately 1,000 C. elegans embryos on each 10 cm plate to the young adult stage. This sample size avoids overcrowding and starvation, and guarantees enough total RNA for sequencing library construction. We did not get triplicates for each of the 249 strains. A few harvested samples showed low quality or quantity during RNA isolation or sequencing library construction. We performed RNA-seq on all the strains with at least two high-quality replicates.
Data exclusions	Transcripts with unreliable expression and outlier samples were excluded. Please find details in the methods of the manuscript.
Replication	Two to three biological replicates were prepared for each strain. Most intra-strain distances among replicates were smaller than the median of inter-strain distances, demonstrating the reproducibility of results. Please find details in the methods of the manuscript.
Randomization	We randomized samples during animal cultures, RNA extraction, and RNA sequencing.
Blinding	Raw sequencing data were processed through scripts and pipelines.

Reporting for specific materials, systems and methods

We require information from authors about some types of materials, experimental systems and methods used in many studies. Here, indicate whether each material, system or method listed is relevant to your study. If you are not sure if a list item applies to your research, read the appropriate section before selecting a response.

Materials & experimental systems

n/a	Involved in the study	
☒	☐	Antibodies
☒	☐	Eukaryotic cell lines
☒	☐	Palaeontology and archaeology
☐	☒	Animals and other organisms
☒	☐	Human research participants
☒	☐	Clinical data
☒	☐	Dual use research of concern

Methods

n/a	Involved in the study	
☒	☐	ChIP-seq
☒	☐	Flow cytometry
☒	☐	MRI-based neuroimaging

Animals and other organisms

Policy information about studies involving animals; ARRIVE guidelines recommended for reporting animal research

Laboratory animals	Caenorhabditis elegans / hermaphrodite / young adult
Wild animals	The study did not involve wild animals.

Field-collected samples	All 207 strains were obtained from Caenorhabditis elegans Natural Diversity Resource (CeNDR) (https://www.elegansvariation.org/). Animals were cultured at 20°C on modified nematode growth medium (NGMA) containing 1% agar and 0.7% agarose to prevent burrowing and fed Escherichia coli strain OP50.
Ethics oversight	No ethical approval or guidance required for invertebrate animals.

Note that full information on the approval of the study protocol must also be provided in the manuscript.
